# Effect of High‐Speed Shaking on Oxygen Transfer in Shake Flasks

**DOI:** 10.1002/biot.70013

**Published:** 2025-04-14

**Authors:** Andreas Schulte, Andreas Jordan, Wolf Klöckner, Mathias Schumacher, Burkhard Corves, Jochen Büchs

**Affiliations:** ^1^ AVT – Biochemical Engineering RWTH Aachen University Aachen Germany; ^2^ IGMR RWTH Aachen University Aachen Germany

**Keywords:** high‐speed shaking, *Kluyveromyces lactis*, liquid distribution, oxygen transfer rate, shake flask

## Abstract

Shake flasks are predominantly used in screening and the early stages of biotechnological process development. However, oxygen‐demanding processes cannot easily be performed in shake flasks, since the maximum oxygen transfer capacity is usually smaller than in stirred reactors. Studies during the last decades suggest that the shaking frequency is one of the most crucial cultivation parameters to sustainably increase oxygen supply in orbitally shaken bioreactors. In this study — for the first time — a prototype of a self‐balancing orbital shaker was used, which is capable to be operated at up to 750 rpm shaking frequency at 25 mm shaking diameter and 600 rpm at 50 mm. *Kluyveromyces lactis* cultivations were monitored with a modified TOM system to measure the maximum oxygen transfer capacities (OTR_max_) and corresponding *k_L_a* values. A maximum *k_L_a* value of 650 h^−1^ (OTR_max_ = 135 mmol/L/h) was reached at 10 mL filling volume in a 250 mL shake flask made of glass with a hydrophilic surface property. This is an increase of about 50%, compared to current commercial orbital shakers. The new high‐speed orbital shaker provides new possibilities for screening applications and process development. High‐speed shaking for enhanced oxygen supply is particularly beneficial at 25 mm shaking diameter, rather than at 50 mm, minimizing the impact of the elevated centrifugal force on the shaking system.

## Graphical Abstract and Lay Summary

Oxygen supply can be a pitfall in shake flask cultivations that can lead to suboptimal conditions in screening and process development. In this study, high‐speed shaking is applied to shake flasks leading to about 50% increase in *k*_*L*_ *a* compared to conventional shaking. This way the gap between shake flasks and stirred bioreactors can be closed.

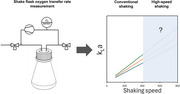

**Abbreviations**
*a*
: volumetric mass transfer area (m^2^/m^3^)
*c*_O2,g_
: mole fraction of oxygen in gas phase (mmol/mmol)
CTR: carbon dioxide transfer rate (mmol/L/h)
*d*_0_
: shaking diameter (m)
*k*_*L*_
: liquid side mass transfer coefficient (m/h)
*k*_*L,bulk*_
: liquid side mass transfer coefficient of the bulk liquid (m/h)
*k*_*L,film*_
: liquid side mass transfer coefficient of the liquid film (m/h)
*k*_*L*_ *a*
: volumetric mass transfer coefficient (h^−1^)
*L*_O2_
: oxygen solubility (mmol/L)
*n*
: shaking frequency (h^−1^)
OTR: oxygen transfer rate (mmol/L/h)
OTR_max_
: maximum oxygen transfer capacity (mmol/L/h)
*V*_L_
: liquid volume (m^3^)

## 1 Introduction

Oxygen supply is a crucial parameter for aerobic biological processes and one of the most critical scale‐up parameters [1]. Insufficient oxygen supply might lead to reduced metabolic activity and may cause the formation of unwanted anaerobic byproducts, reduced product quantity, and quality [2]. Therefore, oxygen consumption is usually monitored in aerobic processes in stirred tank reactors [3]. Oxygen availability is controlled, e.g., by adjusting the aeration rate, stirrer speed, or stirrer type [1]. Further methods to improve the oxygen availability include an increased reactor pressure or aeration with oxygen‐enriched air [4, 5, 6]. However, screening and media optimization are usually conducted in shaken microplates and shake flasks [7, 8], where the above‐mentioned methods can only be applied to a certain extent. Often, too little attention is paid to the oxygen supply. This may lead to wrong decisions during screening which cannot be corrected later [3, 9]. For shaken cultivations, there are several options to improve oxygen supply as follows: (1) baffled shake flasks to increase turbulence and mass transfer area, (2) reduction of the liquid volume, (3) increase of reactor diameter, (4) increase of shaking diameter, or (5) increase of shaking frequency. However, baffled shake flasks can promote out of phase shaking conditions. In this case, the culture broth cannot follow the shaker movement anymore, which in turn leads to reduced power input and oxygen transfer [10]. In addition, baffled shake flasks suffer from low reproducibility [9] and may provoke foam formation as well as wetting and clogging of the sterile barrier with droplets [11]. A reduction of the liquid volume or an increase of the reactor diameter, both aim at increasing the volumetric mass transfer area. However, both methods are limited, as the effect of evaporation is enhanced [12]. Another approach has recently been presented where a concentric glass ring inserted at the bottom of a cylindric glass vessel was used to increase the gas–liquid mass transfer area [13]. Zhu et al. presented the introduction of a hollow but closed cylinder in the middle of an orbitally shaken bioreactor to improve mixing and *k_L_a* [14, 15]. Increasing the shaking diameter or shaking frequency is the only option to improve the oxygen transfer of cultivations in standard shake flasks without adverse effects on the cultivation. However, since the impact of the shaking diameter is relatively small [16, 17], increasing the shaking frequency remains as the only valid and feasible option.

During the last decades, many empirical correlations were developed that describe the volumetric mass transfer coefficient (*k_L_a*) or the maximum oxygen transfer capacity (OTR_max_) in shake flasks as a function of crucial shaking and cultivation parameters (liquid volume *V*_L_, shaking diameter *d*_0_, shaking frequency *n*, shake flask diameter *d*). Meier et al. [17] reviewed these correlations and derived a universal correlation for shake flasks made of glass with liquids with waterlike viscosity that also includes the effect of media composition. However, this correlation was established in an experimental space < 450 rpm shaking frequency and may, therefore, not be extrapolated beyond this point. The only fully mechanistic model for oxygen supply in shake flasks has been developed by Maier and Büchs [18] and Büchs et al. [19].

Though increasing the shaking frequency is the best way to enhance oxygen availability, this option is usually limited by the shaking machine. Manual weight balancing of orbital shakers is laborious and in general imperfect. In 1970, Freedman [20] reported shaking at 800 rpm at 1 in. (25.4 mm) shaking diameter with a modified dynamically balanced shaker for oxygen absorption in a sulfite oxidation reaction. However, to date, the manufacturer's limit in shaking frequency is usually 400 rpm at 25 mm [21, 22, 23].

In this study, a prototype of a self‐balancing orbital shaker (Patent DE 102014111236) is used to apply, to our knowledge, the highest centrifugal force and highest *k_L_a* in a shake flask cultivation [24]. *Kluyveromyces lactis* was cultivated at up to 750 rpm at 25 mm shaking diameter and up to 600 rpm at 50 mm shaking diameter. The OTR_max_ was determined with an inhouse built Transferrate Online Monitoring (TOM) device [25] and results are compared to mechanistic model predictions.

## 2 Materials and Methods

### 2.1 Medium and Cultivation

**FIGURE 1:**
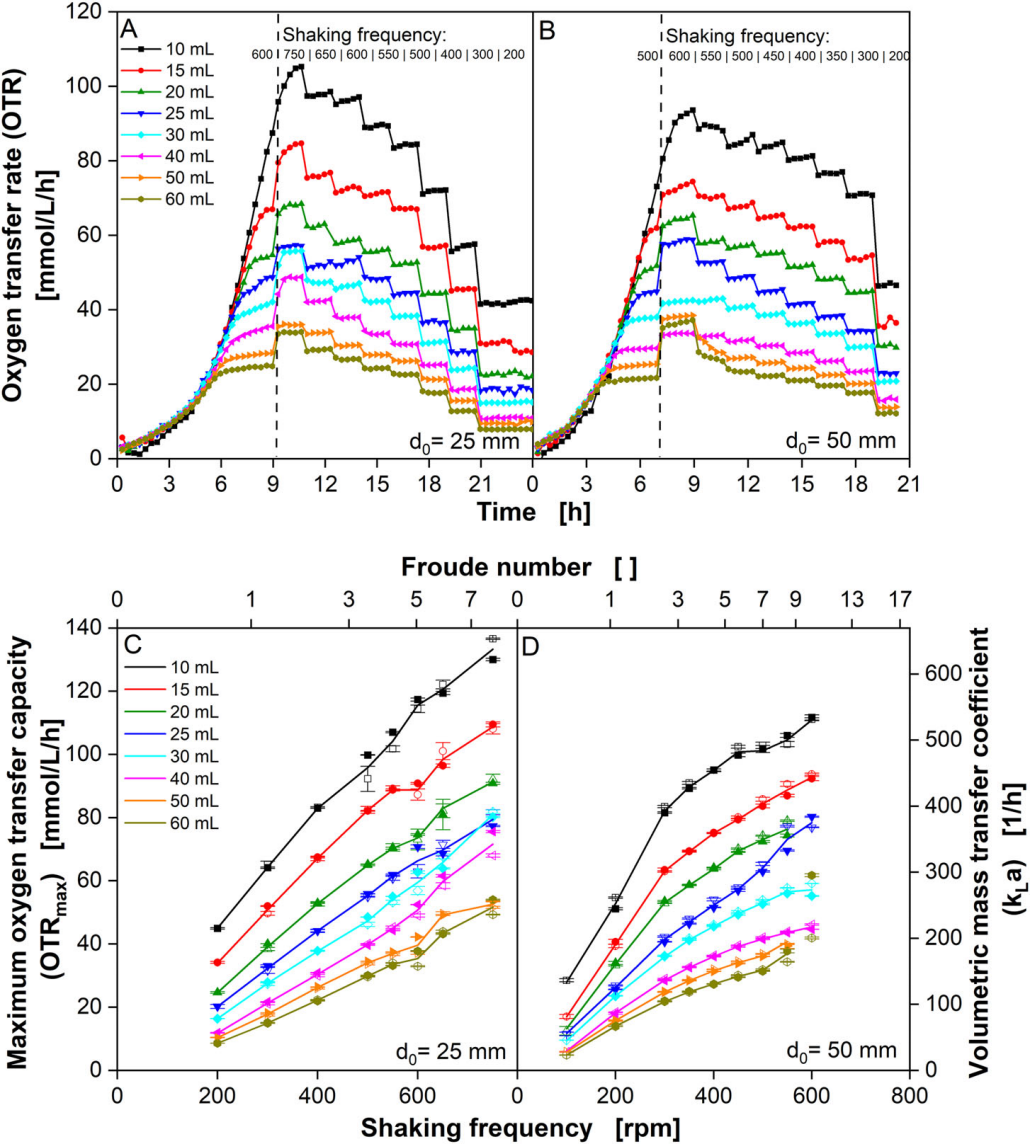
Determination of maximum oxygen transfer capacity (OTR_max_) and volumetric mass transfer coefficient (*k_L_a*) at varying shaking frequencies and filling volumes. (A) and (B): Oxygen transfer rate of *K. lactis* cultivations. Cultivation conditions: YEP Medium (80 g/L glucose), 30°C, 250 mL shake flask, 10–60 mL filling volume, (A) *d*_0_ = 25 mm shaking diameter, variation of the shaking frequency from 750 to 200 rpm, (B) *d*_0_ = 50 mm shaking diameter, variation of the shaking frequency from 600 to 200 rpm. *K. lactis* was cultivated at (A) 600 rpm and (B) 500 rpm shaking frequency, respectively, until the dashed vertical lines. The shaking frequency was then increased to its maximum value and stepwise decreased every 100 min. Every 20 min an OTR measurement was done. The three latest data points (of five points) of each shaking frequency step were considered for subsequent calculations. (C) and (D) The maximum oxygen transfer capacities are determined from *K. lactis* cultivations presented in (A) and (B) (closed symbols) and Supplementary file 1 (open symbols). Data points are an average of three measurements. Standard deviations are presented as error bars. The line connects the average of the closed and open symbols (average of 2 × 3 measurements). The shaking frequency is presented on the bottom *X*‐axis, and the corresponding Froude number (representing the centrifugal acceleration, see Equation 2) is presented on the top *X*‐axis. Cultivation conditions: YEP‐Medium (80 g/L glucose), 30°C, 250 mL shake flask, 10–60 mL filling volume, (C) *d*_0_ = 25 mm shaking diameter, and (D) *d*_0_ = 50 mm shaking diameter. *k_L_a* was calculated from OTR_max_ for an oxygen solubility *L*_O2_ = 0.2093 mmol/L [26] and an oxygen gas mole fraction *C*_O2,g_ = 0.2095 mol/mol according to Equation (1).

In this study, *K. lactis* GG79 pKlac1 was cultivated in YEP medium (Yeast Extract Peptone medium). The yeast strain was kindly provided by the Institute for Molecular Biotechnology of RWTH Aachen University (Germany). The complex YEP medium consisted of 10 g/L yeast extract (lot number 375233217, Roth), 20 g/L peptone/tryptone (lot number: 435235212, Roth). pH was adjusted to 4.8 with 5 M KOH. The pre‐culture medium contained 20 g/L glucose and the main culture 80 g/L glucose as carbon source. Prior to cultivation, ampicillin was added to a final concentration of 0.1 g/L. For the pre‐culture, *V*_L_ = 10 mL of medium was inoculated with 25 µL of cryo‐culture containing 150 g/L glycerol. The pre‐culture was cultivated in a 250 mL RAMOS shake flask overnight at 30°C until an OTR of 50 mmol/L/h was reached in the exponential growth phase. The shaking frequency of the preculture was *n* = 600 rpm at *d*_0_ = 25 mm shaking diameter and *n* = 500 rpm at *d*_0_ = 50 mm. The main culture was inoculated with an optical density (OD_600_) of 0.3, if not otherwise stated. The main culture was cultivated at 30°C in a 250 mL RAMOS shake flask at *V*_L_ = 10, 15, 20, 25, 30, 40, 50, and 60 mL. Main cultures at *d*_0_ = 25 mm shaking diameter were started at *n* = 600 rpm until all cultivations left the exponential growth phase. The shaking frequency was then increased to *n* = 750 rpm and subsequently stepwise decreased every 100 min. Main cultures at *d*_0_ = 50 mm shaking diameter were started at *n* = 500 rpm until all cultivations left the exponential growth phase. The shaking frequency was then increased to *n* = 600 rpm and subsequently stepwise decreased every 100 min (Figures 1A,B and S1).

### 2.2 Measurement Setup

The oxygen transfer rates of eight parallel shake flask cultivations were determined with an in‐house built TOM (Transferrate Online Monitoring) device [27] based on the RAMOS technology according to Anderlei et al. [25]. In the TOM device, the shake flasks (250 mL nominal volume) are aerated at a flow of 10 smL/min (11.1 mL/min at 30°C), according to the ventilation in 250 mL Erlenmeyer narrow neck flasks with cotton plugs [28]. Standard 250 mL shake flasks with a threaded neck were used. No geometrical change was done to the part of the flask which is in contact with the rotating liquid. An electrochemical oxygen sensor is used to measure the oxygen partial pressure in the shake flask headspace. In recurring measurement phases, the aeration is stopped (15 min aeration, 5 min measurement phase). The OTR is calculated from the slope in oxygen partial pressure during these measurement phases.

### 2.3 High‐Speed Shaker

An LS‐X orbital shaker from Adolf Kühner AG (Birsfelden, Switzerland) was used for high‐speed orbital shaking experiments. The shaker was modified in cooperation with the Institute of Mechanism Theory, Machine Dynamics and Robotics (IGMR) at RWTH Aachen University. The shaker was equipped with a self‐balancing mechanism that is described in Patent DE 102014111236. Unbalanced forces are automatically and passively (without any source of energy or sensors) minimized. Thus, shaking frequencies up to 750 rpm at a shaking diameter of 25 mm and 600 rpm at a shaking diameter of 50 mm could be achieved.

### 2.4 Calculation of Mass Transfer Area and Maximum Oxygen Transfer Capacity by a Mechanistic Model

The mass transfer area between the gas phase and the liquid phase in the shake flask is calculated according to Büchs et al. [19]. One model assumption is a frictionless movement of the liquid on the shake flask wall. Therefore, the model is valid only for waterlike viscous liquids. The viscosity of the culture broth in the present study never exceeded 1.4 mPa s (determined with a cone‐plate rheometer [PhysicaMCR301 Modular, Anton Paar]). The model distinguishes between the mass transfer area of the rotating bulk liquid and the mass transfer area that builds up as hydrophilic liquid film on the shake flask bottom and the shake flask wall. It is named “liquid distribution model” in the following. *k_L_a* and OTR_max_ were calculated according to the “two sub‐reactor model” presented by Maier et al. [18] and using the liquid distribution model according to Büchs et al. [19]. This approach divides the liquid phase in the shake flask into two sub‐reactors. The bulk liquid is modeled as an ideally mixed surface aerated reactor, according to a model derived by Gnielinski [29]. The liquid film on the shake flask wall and bottom is described as a film reactor, according to Higbie's penetration theory [19]. The “two sub‐reactor model” is fully mechanistic and needs no fitting parameters, as typical for bubble aerated reactors. It may, therefore, be applied to predict *k_L_a* and OTR_max_ values at elevated shaking frequencies that were not part of the study by Maier et al. [18]. In this study, only hydrophilic glass flasks were used, where a liquid film forms on the glass wall. In contrast, for plastic flasks, the formation of a liquid film is hindered, due to the hydrophobicity of the surface.

The general equation to calculate *k_L_a* from OTR_max_ and vice versa is presented below:

(1) $$OTR_{\max}=k_{L}\;a\times L_{O2}\times c_{O2,g\left(20.95\%\right)}$$

*c*_O2,g(20.95%)_ is the ambient O_2_ mole fraction. In the shake flask headspace an O_2_ mole fraction < 20.95% is present as the sterile barrier or active aeration (in this case 11.1 mL/min for all filling volumes) limits the air exchange. To account for different filling volumes and aeration rates, the measured maximum OTR values in this study were normalized to an O_2_ mole fraction of 20.95% (theoretical maximum at strong aeration with ambient air) using Equation (2).

(2) $$\begin{array}{cc} OTR_{\max} & =measured\;OTR\left(oxygen\;limited\right)\\ & \;\times \frac{0.2095}{measured\;O_{2}\;mole\;fraction} \end{array}$$

The O_2_ concentration in the liquid is assumed to be closed to 0 mmol/L in the bulk liquid under oxygen limited conditions and is, thus, not represented in Equation (1). The parameters in Table 1 were used to calculate *k_L_a* from measured OTR_max_ data and OTR_max_ from model‐derived *k_L_a* values.

**TABLE 1:** Input parameters for mechanistic model and *k*_*L*_ *a* calculation.

Quantity	Definition	Value	Unit	Source
*η*	Broth viscosity	1.4	mPa s	Self‐determined
*L*_O2_	Oxygen solubility	0.2093	mmol/L	[26]
*D*_O2_	Oxygen diffusion coefficient	1.564 × 10^−5^	cm^2^/s	[30, 31]
*c*_O2,g(20.95%)_	Maximum oxygen gas mole fraction	0.2095	mmol/mmol	

## 3 Results

### 3.1 Maximum Oxygen Transfer Capacity at Elevated Shaking Frequencies Derived From Oxygen Limited Cultivations

The effect of elevated shaking frequencies on oxygen transfer during shake flask cultivations was analyzed during cultivations of the yeast *K. lactis*. In preliminary experiments, *K. lactis* exhibited a fast and exponential growth phase at a glucose concentration of 80 g/L in YEP medium. After reaching oxygen limitation, continuous oxygen‐limited respiration was observed for several hours (*d*_0_ = 25 mm, *V*_L_ = 10 mL, *n* = 600 rpm, OTR ≈ 100 mmol/L/h, 12 h oxygen limitation, data not shown), without being influenced by limiting co‐substrates, pH changes, inhibiting products or by‐products. This phase of oxygen‐limited growth was, therefore, regarded as being suitable for investigating the *k_L_a* and OTR_max_. Flitsch et al. [32] have previously presented respiration data for oxygen‐limited *K. lactis* cultures growing under similar conditions at 40 g/L glucose in YEP medium. Figure 1A,B presents the course of OTR during *K. lactis* cultivations with filling volumes ranging from 10 to 60 mL at *d*_0_ = 25 mm (A) and *d*_0_ = 50 mm (B). The initial phase of the cultivation was conducted at 600 rpm (*d*_0_ = 25 mm) and 500 rpm (*d*_0_ = 50 mm). In this initial part of the cultivation, cultures started growing exponentially, followed by oxygen limitation (15–60 mL filling volume) or just leaving the exponential growth phase (10 mL). The higher the filling volume, the earlier an oxygen limitation occurs and the lower the OTR during oxygen limitation. After this initial growth phase, the shaking frequency was increased to the possible maximum, 750 rpm (*d*_0_ = 25 mm) and 600 rpm (*d*_0_ = 50 mm). Subsequently, the shaking frequency was stepwise lowered every 100 min, resulting in less and less oxygen supply. This way, the cultures were kept oxygen limited throughout the whole cultivation. Clearly, distinguishable OTR plateaus develop at each shaking frequency. The last four measured OTR values in the plateau from each shaking frequency step were averaged and corresponding *k_L_a* and OTR_max_ values were calculated (OTR_max_ is the corresponding OTR at 20.95% oxygen). The same calculations were performed for data from another individual experiment (depicted in Figure S1). Values from both individual experiments showed only minor deviations. Figure 1C,D summarizes the results from the calculations for different shaking frequencies, shaking diameters, and filling volumes. The maximum *k_L_a* reached in this study was 650 h^−1^ at *n* = 750 rpm, *d*_0_ = 25 mm, and *V*_L_ = 10 mL (respective OTR_max_ = 135 mmol/L/h). This is approx. 50% higher than what is possible with commercial shaking machines. It is in the same range as *k_L_a* values achieved with numerous single use stirred tank bioreactors for lab and pilot scale [33]. Similar oxygen transfer rates in a shake flask cultivation have only been shown by Hansen et al. [13]_,_ using cylindrical vessels with concentrically inserted glass rings for increased liquid film surface area.

At *d*_0_ = 50 mm, the OTR_max_ (respective *k_L_a*) steeply increases up to 300 rpm. Above 300 rpm, the increase in OTR_max_ tends to slow down, while for *d*_0_ = 25 mm the correlation between OTR_max_ and shaking frequency shows close to linear behavior. OTR_max_ increases with decreasing filling volume as found in many correlations before [17]. The upper *X*‐axis shows the Froude number (Fr) as parameter, representing the centrifugal force applied to the shake flask (see Equation 2).

(3) $$Fr=\left({\left(2\times pi\times n\right)}^{2}\;\times \;d_{0}\right)\;/\;\left(2\;\times g\right)$$

The highest Froude number reached during this study was roughly 10 at *d*_0_ = 50 mm, which correlates to 10 ×  *g* centrifugal force. The self‐balancing mechanism of the orbital shaker prototype allowed for shaking frequencies and corresponding centrifugal forces that have not been reported so far at 50 mm shaking diameter. However, the highest determined *k_L_a* was 650 h^−1^ at 750 rpm and *d*_0_ = 25 mm (Froude number = 7.86).

Peter et al. [34] suggested a critical Reynolds number of 60,000 for the transition between laminar and turbulent flow regime in a shake flask. In this current study, a maximum Reynolds number of approx. 64,500 at *d*_0_ = 25 mm and 750 rpm was reached and approx. 51,600 at *d*_0_ = 50 mm and 600 rpm. In commercially available incubator shakers, such high Reynolds numbers can only be achieved in flasks with 500 mL nominal volume or bigger.

### 3.2 Model Predicted *k_L_a*

OTR_max_ and *k_L_a* data derived from oxygen limited *K. lactis* cultivations (Figure 1C,D) were compared with predicted OTR_max_ and *k_L_a* values from the mechanistic two sub‐reactor model, presented by Maier et al. [18], combined with the liquid distribution model by Büchs et al. [19]. The model‐predicted *k_L_a* is a composite of the liquid side mass transfer coefficient *k_L_* and the volumetric mass transfer area *a*, which are calculated separately for the rotating bulk liquid and the liquid film on the flask wall.

### 3.3 Total Mass Transfer Area

The total mass transfer area is divided into the bulk liquid area and the liquid film area that forms on the glass wall during shaking. The total mass transfer area calculated from the liquid distribution model by Büchs et al. [19] is presented in Figure S2. It asymptotically moves into a maximum with increasing shaking frequency, due to the conical shape of the shake flask. The maximum liquid height is reached, when the bulk liquid is forming a vertical interface to the flask headspace. In contrast to this, in a cylindrical vessel, the mass transfer area would continue to increase with increasing centrifugal force. This finding was previously mentioned by Maier [35]. The experimental conditions in this study (at *d*_0_ = 25 mm and 750 rpm and at *d*_0_ = 50 mm and 600 rpm) led to calculated total mass transfer areas that were only 4% less than at the theoretical maximum (2000 rpm used as the upper limit for calculation). According to these calculations, shaking frequencies above 400 rpm lead only to a minor increase in mass transfer area (approx. 15% increase from 400 to 750 rpm at *d*_0_ = 25 mm). This increase in total volumetric mass transfer area can mainly be attributed to the increase in liquid film area as the bulk liquid area remains rather constant within the simulated range (see Figure S3). When the total mass transfer area comes close to its maximum (above 400 rpm), the mass transfer coefficient *k_L_* of the bulk liquid (*k_L,bulk_*) and the liquid film (*k_L,film_*) as well as the liquid distribution (the distribution between bulk liquid area and liquid film area) must be the reason for increasing *k_L_a* values at increasing shaking frequency.

### 3.4 Liquid Distribution and *k_L_* Values

**FIGURE 2:**
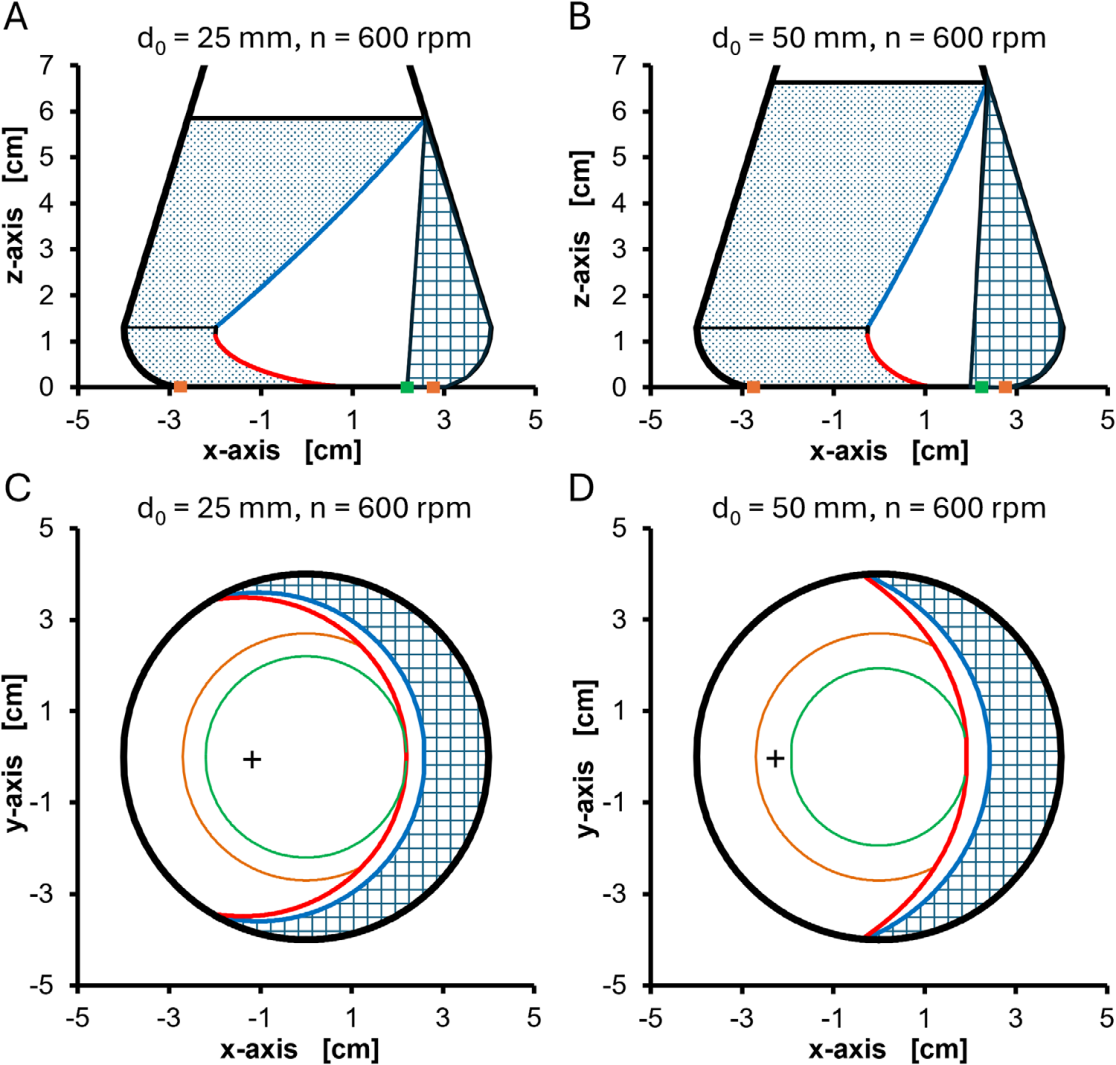
Calculated liquid distribution (as presented by Büchs et al. [19]) in a 250 mL shake flask at the same shaking frequency but different shaking diameters. Operating conditions: 600 rpm shaking frequency, 250 mL shake flask, 50 mL filling volume (A) side view, *d*_0_ = 25 mm shaking diameter, (Fr = 5.03) (B) side view, *d*_0_ = 50 mm shaking diameter, (Fr = 10.06) (C) top view, *d*_0_ = 25 mm shaking diameter (D) top view, *d*_0_ = 50 mm shaking diameter. The contact line between the bulk liquid and the glass wall is represented as the blue line in the conical upper part of the shake flask and as the red line in the quarter torus. The green circle represents the minimum diameter that is wetted by the rotating liquid and the orange circle marks the transition from the flat bottom to the quarter torus of the shake flask. The black square marks the center of the orbital shaking movement.

Figure 2 exemplarily shows the calculated liquid distribution for both shaking diameters at *n* = 600 and *V*_L_ = 50 mL. The area between red line, blue line, and the flask wall on the right‐hand side marks the bulk liquid, while the dotted area represents the shake flask glass wall covered with a liquid film. For *d*_0_ = 50 mm, compared to *d*_0_ = 25 mm, the maximum liquid height at 600 rpm is higher and the bulk liquid covers a significantly smaller angle of the flask perimeter. This pattern of liquid distribution is found for the whole simulated range of shaking frequency (100–800 rpm) and filling volume (10–60 mL) and is caused by the higher centrifugal force and the more shifted center of the orbital shaking movement relative to the flask center at *d*_0_ = 50 mm, compared to *d*_0_ = 25 mm (see black cross in Figure 2C,D). The higher liquid height at *d*_0_ = 50 mm explains the aforementioned larger total mass transfer area (bulk liquid + liquid film) at *d*_0_ = 50 mm. However, the mass transfer area distribution (see Figure S3) shows that only the liquid film area is larger at *d*_0_ = 50 mm, while the bulk liquid area is larger for *d*_0_ = 25 mm.

According to the two sub‐reactor model by Maier et al. [18], the mass transfer coefficient for the bulk liquid (*k_L,bulk_*) increases quasi‐proportional with shaking frequency, while the correlation is sub‐proportional for the liquid film (*k_L,film_*) (see Figure S4). For both, *k_L,bulk_* and *k_L,film_*, no major differences are predicted by the two sub‐reactor model between *d*_0_ = 25 mm and *d*_0_ = 50 mm, indicating that shaking diameter is subordinate for the *k_L_* value, compared to shaking frequency. For all shaking frequencies simulated for this study, *k_L,film_* is always larger than *k_L,bulk_*, supporting the importance of the liquid film for mass transfer [36].

Summing up, the bulk liquid area, the liquid film area, and the *k_L,film_* are increasing sub‐proportional with shaking frequency, while the *k_L,bulk_* is increasing quasi‐proportional with shaking frequency. Consequently, the resulting total *k_L_a*, predicted by the two sub‐reactor model must follow a sub‐proportional trend with shaking frequency (see Equation 3).

(4) $$k_{L}a=k_{L,\mathrm{bulk}}\times a_{\mathrm{bulk}}+k_{L,\mathrm{film}}\times a_{\mathrm{film}}$$

In literature, though, empirical model coefficients for estimating the *k_L_a* as function of shaking frequency (in addition to other parameters) are usually above or equal to 1, e.g., *k_L_a ∼ n* ^1^ [37], *k_L_a ∼ n* ^1.16^ [16], *k_L_a ∼ n* ^1.18‐Osmol/10.1^ [17] (*n* ^1.081^ for 1 osmol/kg). Liu et al. [38] are the only ones suggesting a sub‐proportional correlation (*k_L_a ∼ n* ^0.88^). However, the experimental space of Liu et al. was limited to *n* = 200 rpm and *n* = 250 rpm at *d*_0_ = 25 mm [38]. The saturation in mass transfer area can, therefore, not be the reason for their finding. However, no empirical model was yet developed with datasets exceeding *n* = 450 rpm and may, therefore, not be extrapolated beyond this shaking frequency.

### 3.5 Influence of Froude Number and Shaking Diameter on Oxygen Transfer and Liquid Distribution

**FIGURE 3:**
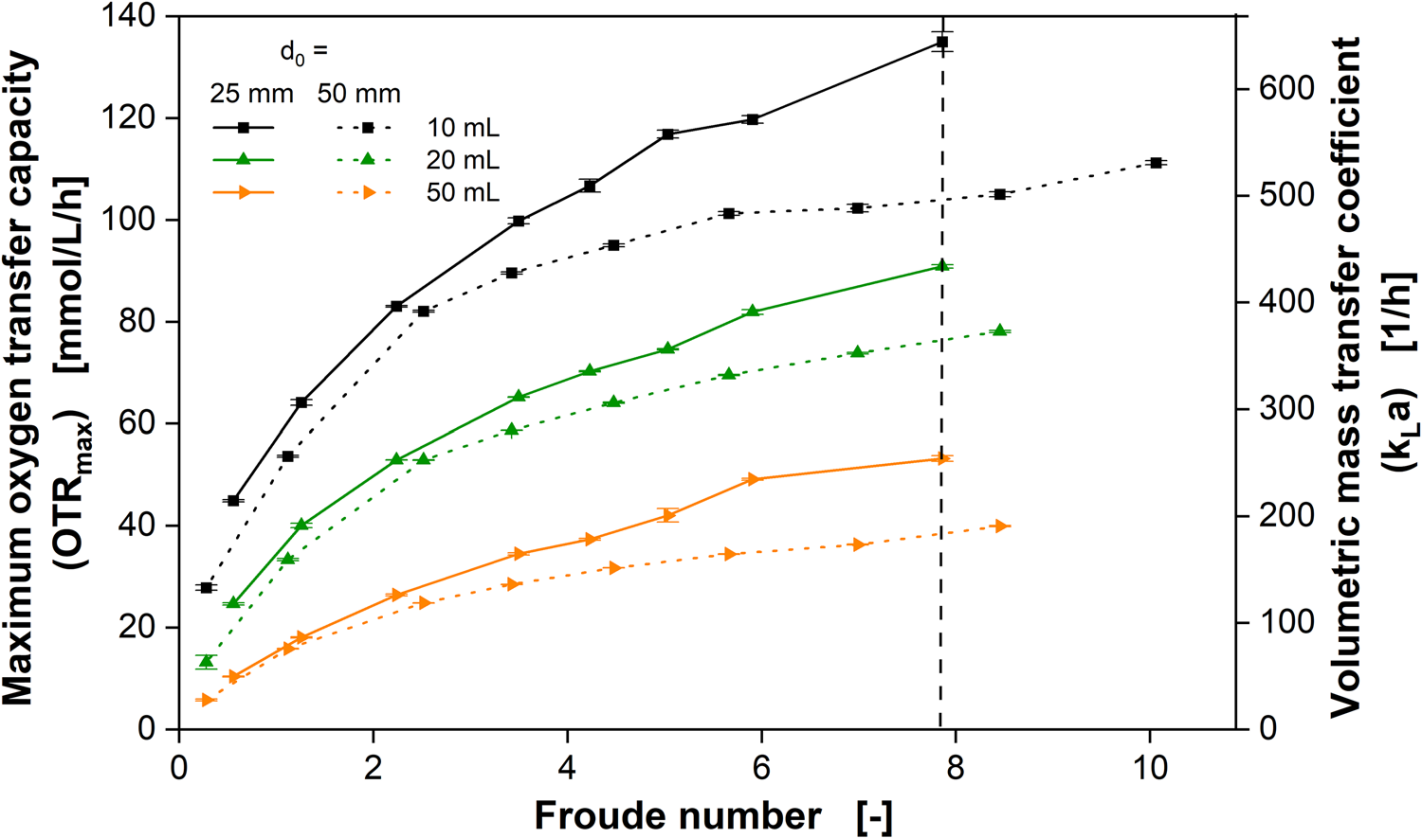
Influence of the Froude number (representing centrifugal acceleration, see Equation 2) and shaking diameter on the maximum oxygen transfer capacity (OTR_max_) and volumetric mass transfer coefficient (*k_L_a*). Data points are an average of two individual experiments presented in Figures 1 and S1. Standard deviations are derived from six measurements from two individual experiments (2 × 3 measurements) and are presented as error bars. For clarity, only data for filling volumes of 10, 20, and 50 mL are shown. Cultivation conditions: YEP‐Medium (80 g/L glucose); 30°C; 250 mL shake flask; 10, 20, and 50 mL filling volume; *d*_0_ = 25 mm shaking diameter (solid lines) and *d*_0_ = 50 mm shaking diameter (dashed lines). The vertical black dashed line is marking Fr = 7.86, which corresponds to *d*_0_ = 25 mm and 750 rpm and *d*_0_ = 50 mm and 530 rpm.

Figure 3 illustrates the OTR_max_ and *k_L_a* as function of the Froude number for *d*_0_ = 25 mm and *d*_0_ = 50 mm. The data presented in Figure 3 were derived from the experimental datasets shown in Figures 1 and S1. Only three different filling volumes are shown for clarity of the figure. The Froude number embodies the centrifugal force that acts on the shake flask and the whole shaking system. Not carefully balanced shaking machines exhibit stronger vibrations and mechanical wear at elevated levels of the centrifugal force. Therefore, it is favorable to run a shaker at a low Froude number, when applicable. When the same Froude number (same centrifugal force) is applied at *d*_0_ = 25 mm and *d*_0_ = 50 mm, the maximum oxygen transfer capacity is higher at *d*_0_ = 25 mm (see Figure 3) in the whole experimental range of this study (100–750 rpm). This conclusion could already be drawn from current *k_L_a* correlations [16, 17] up to 450 rpm and has been shown experimentally by Akgün et al. [39] for shaking frequencies up to 325 rpm.

The correlation between oxygen transfer and Froude number is clearly sub‐proportional for both shaking diameters. Therefore, the increase of the oxygen transfer is increasingly smaller at higher Froude numbers. Figure S5 is presenting the liquid distribution at Fr = 7.86 (marked as vertical dashed line in Figure 3) exemplarily for 50 mL filling volume. While the centrifugal force is the same in both cases (*d*_0_ = 25 mm and *d*_0_ = 50 mm), the liquid distribution strongly differs. The liquid height and, consequently, the total mass transfer area are slightly larger for *d*_0_ = 50 mm. At *d*_0_ = 25, the liquid film area covers about 60% (40% bulk liquid area) of the total mass transfer area while at *d*_0_ = 50 the liquid film covers about 70% (30% bulk area) (compare solid [*d*_0_ = 25 mm] and dashed [*d*_0_ = 50 mm] circles in Figure S3B). Applying the same centrifugal force, the superficial velocity of the rotating liquid is higher at *d*_0_ = 25 mm than at *d*_0_ = 50 (in this case 750 rpm compared to 530 rpm). Thus, at *d*_0_ = 25 mm, the recovery of the liquid film occurs more often, the contact time between liquid film and headspace is shorter and the mass transfer coefficient *k_L_* of the bulk liquid and the liquid film is higher due to the higher superficial velocity (compare solid [*d*_0_ = 25 mm] and dashed [*d*_0_ = 50 mm] circles in Figure S4B).

To minimize mechanical stress on the shaking machines or improve oxygen transfer at the same mechanical stress, a small shaking diameter may be recommended. However, when choosing a smaller shaking diameter, it has to be taken into account that this may favor out of phase operation conditions, especially for viscous cultivation broths [40].

### 3.6 Comparison of Model Data and Experimental Results

**FIGURE 4:**
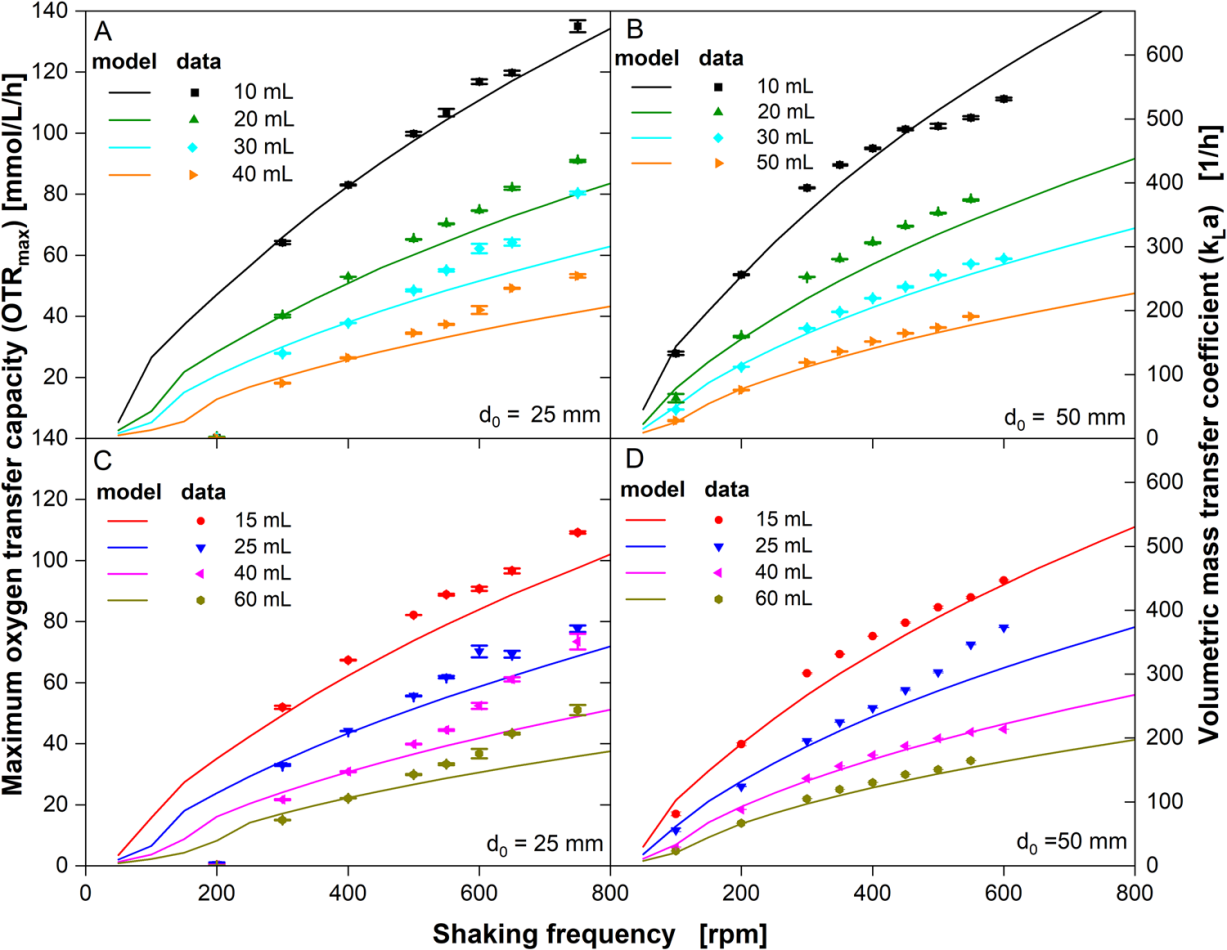
Comparison of maximum oxygen transfer capacity (OTR_max_) measurements and volumetric mass transfer coefficient (*k_L_a*) with mechanistic model data. Data points are an average of two individual experiments, presented in Figure 1C,D. Standard deviations are derived from six measurements from two individual experiments (2 × 3 measurements) and are presented as error bars. The mechanistic model for the determination of the maximum oxygen transfer capacity is described by Maier et al. [18] and Büchs et al. [19]. Data derived from the model are presented as solid lines. The corresponding *k_L_a* is presented on the right *Y*‐axis. For better visibility, filling volumes are depicted alternating in the upper and lower graphs. Cultivation conditions: YEPMedium (80 g/L glucose), 30°C, 250 mL shake flask, 10–60 mL filling volume, (A and C) *d*_0_ = 25 mm shaking diameter, (B and D) *d*_0_ = 50 mm shaking diameter.

Figure 4 compares model predicted (two sub‐reactor model [18]) and experimentally measured OTR_max_ and *k_L_a*, derived from the datasets in Figures 1 and S1. For *d*_0_ = 25 mm, the measured oxygen transfer is well‐predicted by the model (solid lines) for filling volumes up to 25 mL (Figure 4A,C). For filling volumes from 30 to 60 mL, the experimental values follow a more linear than sub‐proportional trend leading to a deviation from the model predicted values above 500 rpm shaking frequency (light blue and orange data points in Figure 4A, pink and kaki data points in Figure 4C). For *d*_0_ = 50 mm, the model well predicts the sub‐proportional increase in oxygen transfer with increasing shaking frequency (Figure 4B,D).

**FIGURE 5:**
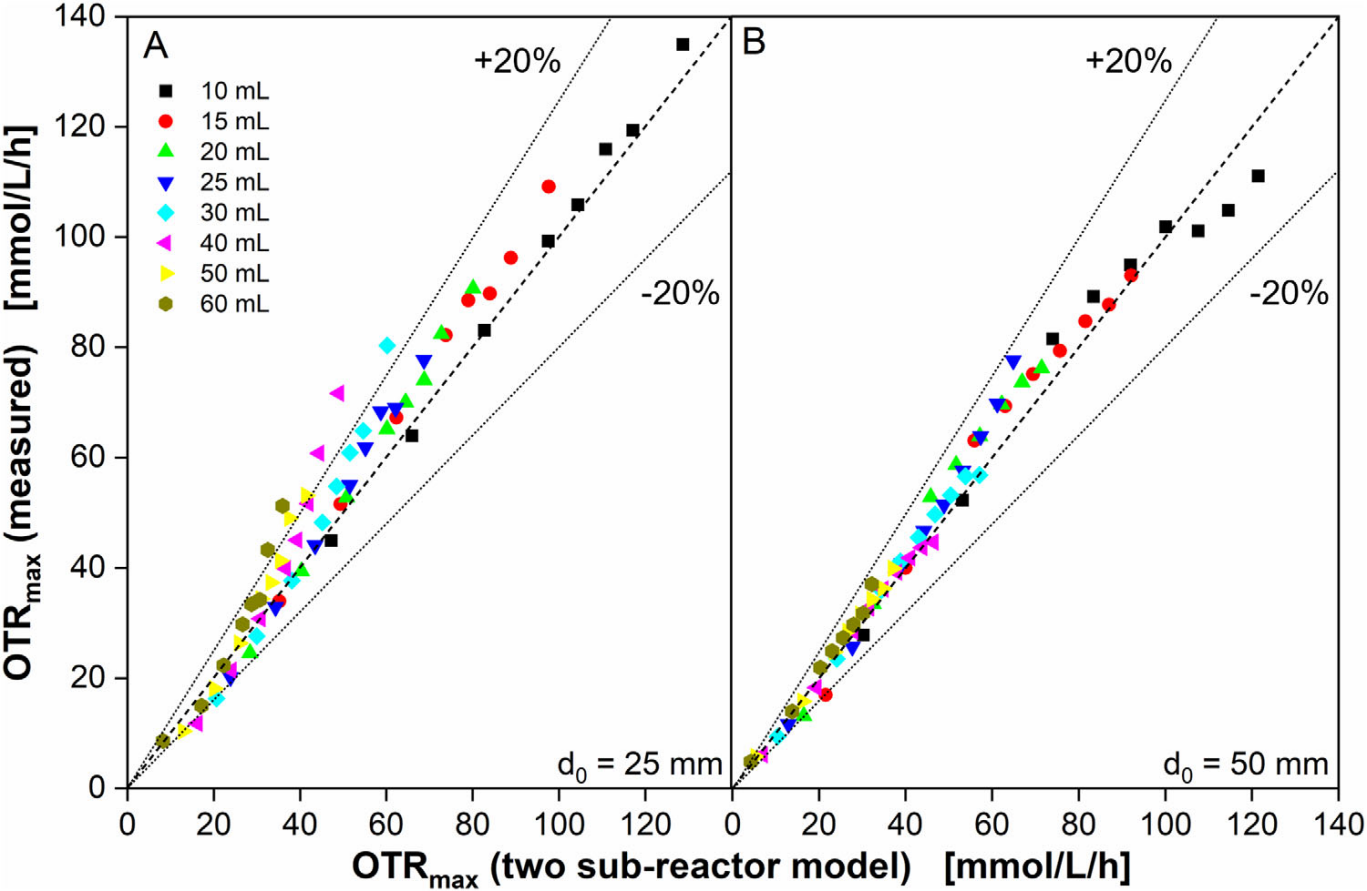
Parity plot of modeled and measured OTR_max_. Data points are derived from Figure 4. Measured OTR_max_ is an average of six measurements from two individual experiments (2 × 3 measurements). Cultivation conditions: YEPMedium (80 g/L glucose), 30°C, 250 mL shake flask, 10–60 mL filling volume, (A) *d*_0_ = 25 mm shaking diameter, (B) *d*_0_ = 50 mm shaking diameter. The dotted lines are marking a range of ± 20% deviation between experimental data and model prediction.

The prediction quality is in general remarkable, as the model is fully mechanistic and does not use any fitting parameter to be adapted. The parity plot of modeled and measured OTR_max_ in Figure 5 demonstrates that the error is mostly < ± 20%. Possible reasons for partial deviations between model predicted data and experimental data (filling volumes between 30 and 60 mL at *d*_0_ = 25 mm) should be discussed briefly. The cultivation broth in this study maintained a viscosity of approx. 1.4 mPa s throughout the cultivation. Ottow et al. [41] as well as Azizan and Büchs [42] have shown that friction at waterlike viscosities leads to a slightly unsymmetrical liquid distribution, which is not taken into account by the liquid distribution model [19]. Their findings are supported by CFD studies performed by Dinter et al. [43, 44]. The liquid distribution may, therefore, be one reason for the deviation between model predicted data and experimental data under some conditions. Also, oxygen solubility and oxygen diffusivity are input parameters of the model [18]. These were estimated according to current literature, based on media composition and temperature [26, 30, 31]. Changes in media composition may influence these parameters (as suggested by Flitsch et al. [32]) but were not considered in this study. However, the cultivation progress was very much alike for both shaking diameters (compare Figure 1A,C). Comparative results between *d*_0_ = 25 mm and *d*_0_ = 50 mm should, therefore, not be influenced. Further investigation of deviation between the model and experimental data at high shaking frequencies and high filling volume should be conducted in future studies.

## 4 Conclusion

An oxygen demanding *K. lactis* shake flask cultivation was successfully performed on a new high‐speed orbital shaker prototype at 25 and 50 mm shaking diameter. A maximum *k_L_a* of 650 h^−1^ (OTR_max_ = 135 mmol/L/h) at 10 mL filling volume in a 250 mL shake flask at 25 mm shaking diameter and 750 rpm was reached. The experimental findings in this study suggest a slightly sub‐proportional correlation between shaking frequency, *k_L_a*, and respective OTR_max_ at shaking frequencies up to 750 rpm, while current empirical correlations (ranging up to 450 rpm) suggest an over‐proportional correlation. The mechanistic two sub‐reactor model presented by Maier et al. [18], using the liquid distribution model presented by Büchs et al. [19], describes well the experimentally observed relation between oxygen transfer and shaking frequency, as it considers a saturation in mass transfer area and a sub‐proportional increase in the liquid side mass transfer coefficient (*k_L_*_,*film*_) of the liquid film that is formed on the hydrophilic shake flask wall. However, for the smaller shaking diameter of 25 mm the *k_L_a* is slightly underestimated at high filling volumes (30–60 mL). In the future, a comparison of this finding with results from CFD analysis should be performed. It was found that the *k_L_a* and respective OTR_max_ was always higher at *d*_0_ = 25 mm compared to *d*_0_ = 50 mm, if the same Froude number was applied. For oxygen‐demanding processes, a smaller shaking diameter may, therefore, be the better trade‐off between oxygen supply and vibration and machine wear.

High speed shaking can substantially increase the *k_L_a* in shake flasks, compared to currently available shaking machines. *K_L_a* is increased without requiring special experimental procedures, such as oxygen enrichment, change of shake flask shape, going to very low filling volumes, or applying large flasks. For applications in industry and research, a commercialization of the used high‐speed orbital shaker prototype would, therefore, be desirable. Safe handling as well as robust and easy to use flask clamping must be addressed in future work.

*K_L_a* values reported in this work are close to those in industrial scale and single use stirred bioreactors [1, 13, 33], while miniaturized bioreactors can still achieve much higher *k_L_a* [1].

To fully close the gap between the level of oxygen supply in shake flasks and in stirred bioreactors, a combination of different concepts may be a feasible strategy, e.g. high‐speed shaking combined with concentric wall interiors [13] and oxygen enrichment.

## Author Contributions

**Andreas Schulte**: Conceptualization, Data curation, Formal analysis, Investigation, Visualization, Writing–original draft. **Andreas Jordan**: Data curation, Formal analysis, Investigation. **Wolf Kloeckner**: Resources. **Mathias Schumacher**: Resources. **Burkhard Corves**: Resources. **Jochen Büchs**: Conceptualization, Supervision, Writing–review and editing.

## Conflicts of Interest

Mathias Schumacher, Wolf Klöckner, Burkhard Corves, and Jochen Büchs are inventors of the patent DE 102014111236. Andreas Schulte and Mathias Schumacher are employed at Kuhner Shaker. Otherwise, the authors declare no financial or commercial conflict of interest.

## Supporting information



Supporting Information

## Data Availability

Data are available upon reasonable request.
