# Supplementary material for: Effect of High‐Speed Shaking on Oxygen Transfer in Shake Flasks
Source: Biotechnol J. 2025 Apr 14;20(4):e70013. doi: 10.1002/biot.70013 (PMC11995246; doi:10.1002/biot.70013)
Supplement: Supplementary file 1 — Supporting Information [file BIOT-20-e70013-s001.docx]

**Supplementary Figure 1** Oxygen transfer rate (OTR) of *K. lactis* cultivations at varying shaking frequencies and filling volumes. Cultivation conditions: YEP Medium (80 g/L glucose), 30°C, 250 mL shake flask, 10 – 60 mL filling volume, (A) d_0_ = 25 mm shaking diameter, variation of the shaking frequency from 750 - 300 rpm, (B) d_0_ = 50 mm shaking diameter, variation of the shaking frequency from 600 - 100 rpm. *K. lactis* was cultivated at (A) 600 rpm and (B) 500 rpm shaking frequency, respectively, until all cultures left the exponential growth phase (dashed vertical line). The shaking frequency was then increased to its maximum value and stepwise decreased every 100 min. The three latest points (of five points) of each shaking frequency step were considered for subsequent investigations.


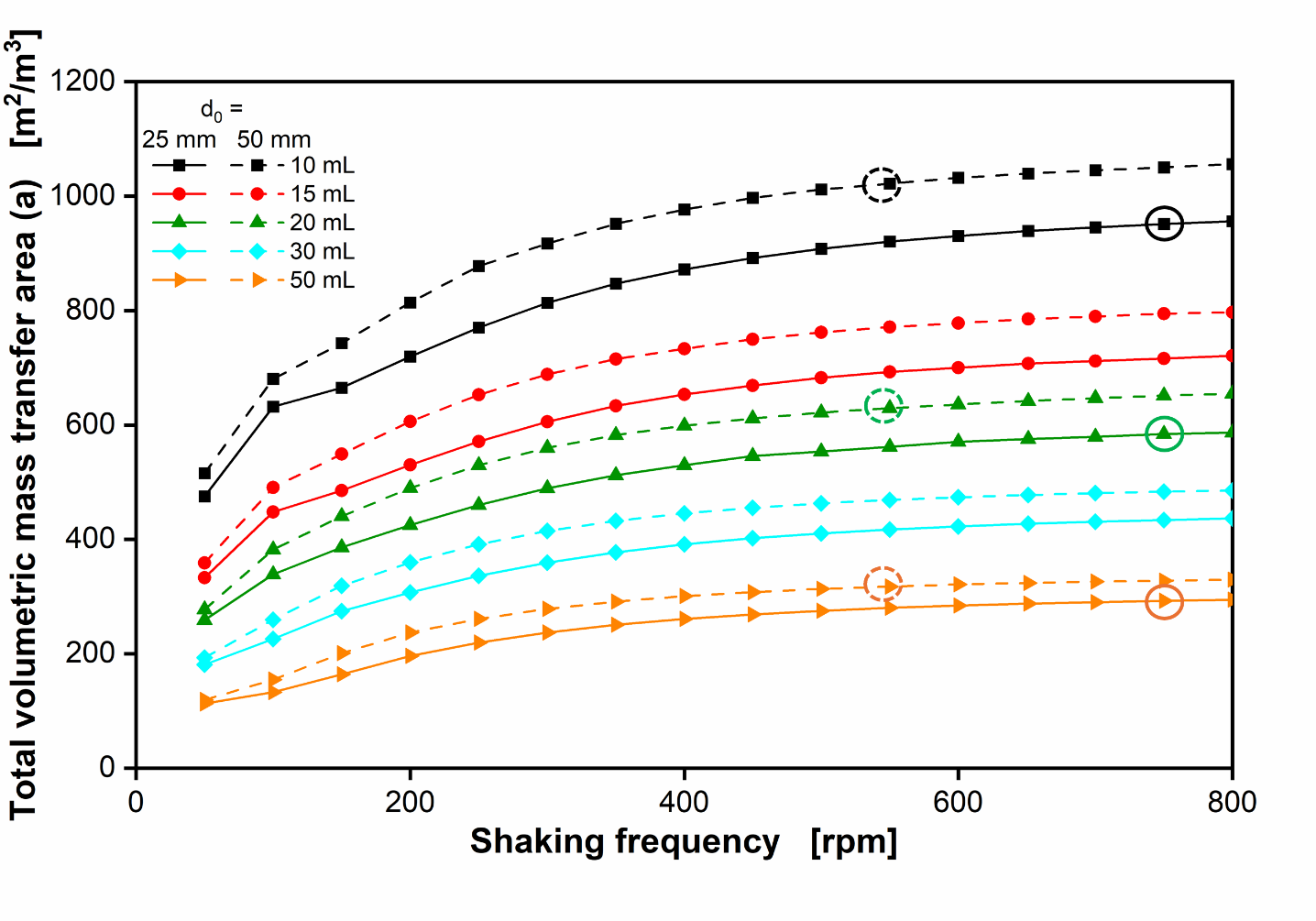


**Supplementary Figure 2** Calculated total volumetric mass transfer areas in a 250 mL shake flask (bulk liquid aera + liquid film area). Operating conditions: d_0_ = 25 mm shaking diameter (solid lines), d_0_ = 50 mm shaking diameter (dashed lines) and varying filling volumes. The solid and dashed circles mark the shaking conditions (750 rpm at d_0_ = 25 mm and 530 rpm at d_0_ = 50 mm) that correspond to the dashed line in Figure 3 and the liquid distribution presented in Supplementary Figure 5 (Froude number = 7.86).


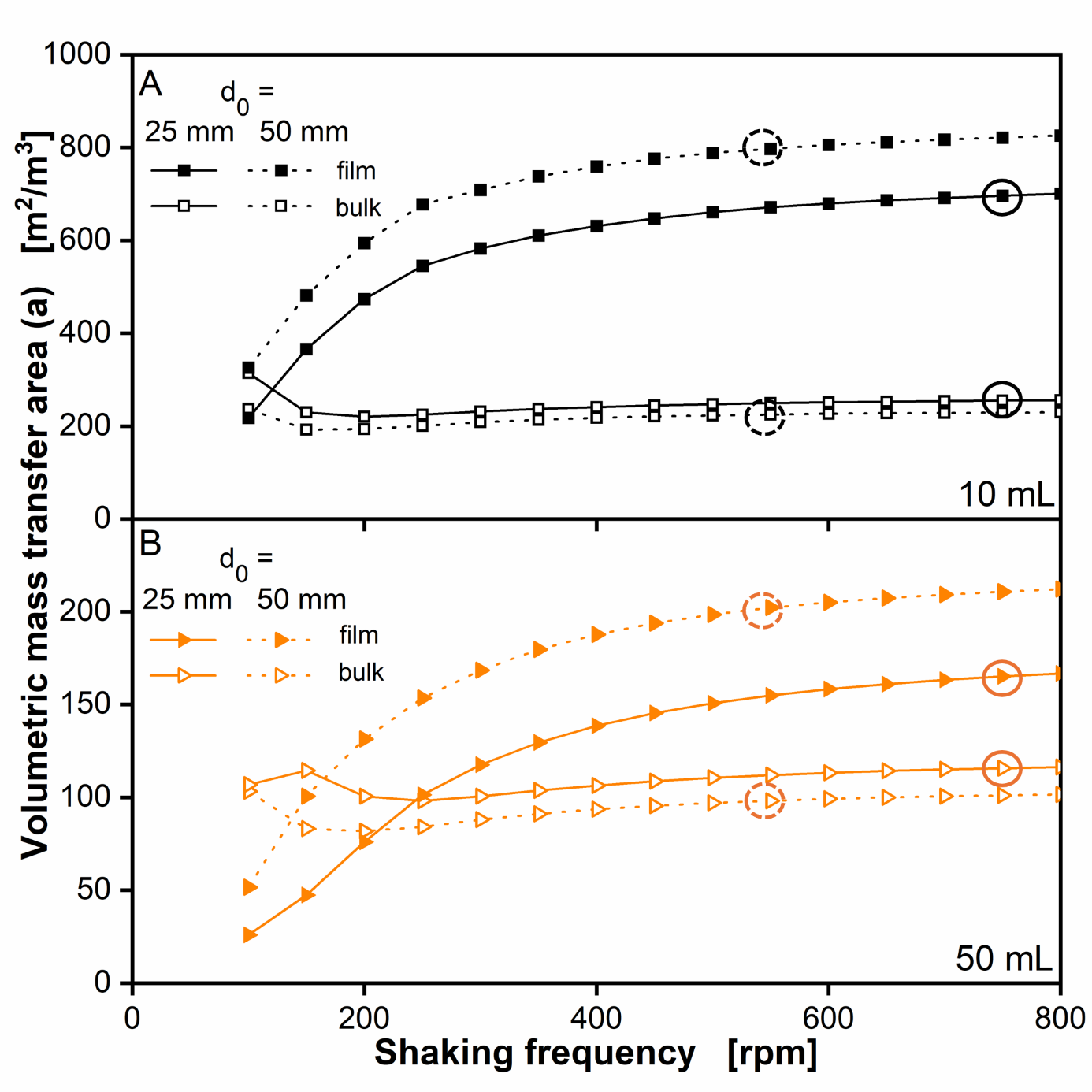


**Supplementary Figure 3** Mass transfer area distribution between bulk liquid and liquid film on the flask wall in a 250 mL shake flask. Operating conditions: d_0_ = 25 mm shaking diameter (solid lines), d_0_ = 50 mm (dashed lines). (A) V_L_ = 10 mL filling volume (black lines) and (B) V_L_ = 50 mL (orange lines) are presented. Bulk liquid area (open symbols) and liquid film area on the flask wall (solid symbols) sum up to the total volumetric mass transfer areas presented in Supplementary Figure 2. The solid and dashed circles mark the shaking conditions (750 rpm at d_0_ = 25 mm and 530 rpm at d_0_ = 50 mm) that correspond to the dashed vertical line in Figure 3 and the liquid distribution presented in Supplementary Figure 5 (Froude number = 7.86).


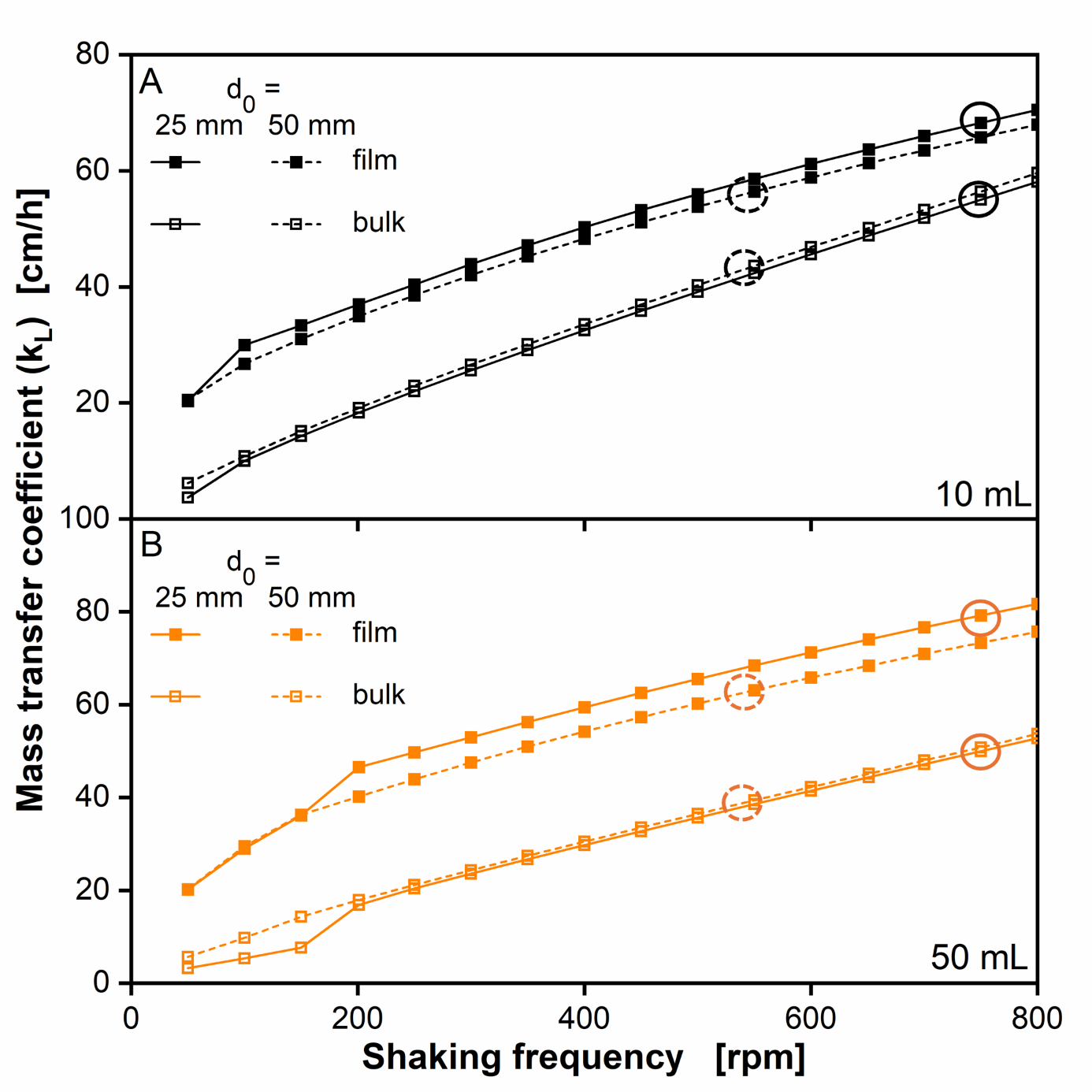


**Supplementary Figure 4** Liquid side mass transfer coefficient at (A) 10 mL filling volume and (B) 50 mL filling volume in a 250 mL shake flask for bulk liquid and the liquid film. Operating conditions: d_0_ = 25 mm shaking diameter (solid lines), d_0_ = 50 mm (dashed lines). *k_L,bulk_* is presented as open symbols and *k_L,film_* as solid symbols. The solid and dashed circles mark the shaking conditions (750 rpm at d_0_ = 25 mm and 530 rpm at d_0_ = 50 mm) that correspond to the dashed vertical line in Figure 3 and the liquid distribution presented in Supplementary Figure 5 (Froude number = 7.86).


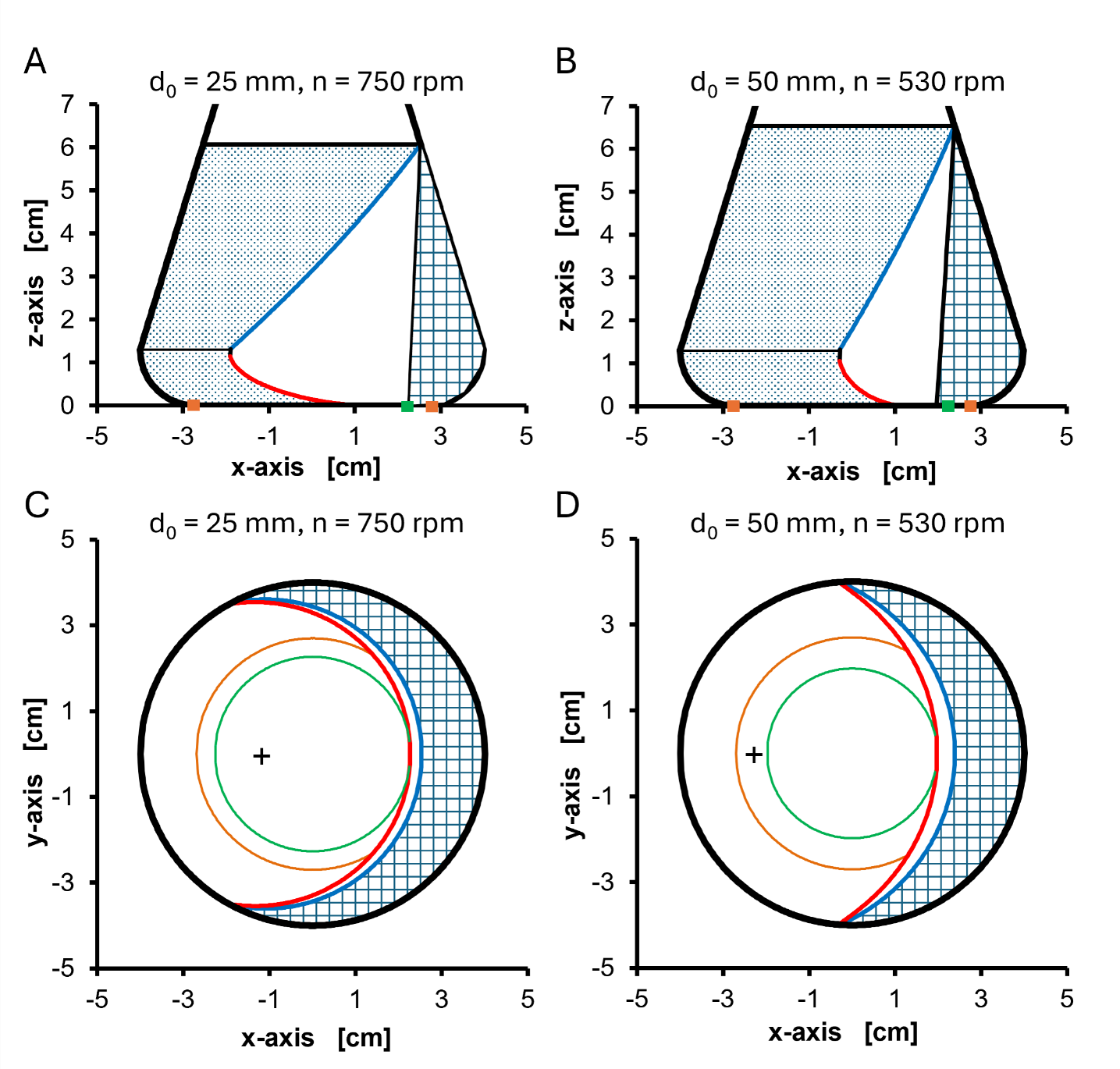


**Supplementary Figure 5** Calculated liquid distribution (as presented by Büchs et al.^[20]^) in a 250 mL shake flask with 50 mL filling volume at the same Froude number, but different shaking diameters (25 and 50 mm). Operating conditions: Froude Number Fr = 7.86 (77 m/s^2^ centrifugal acceleration), (A) side view, 750 rpm shaking frequency, d_0_ = 25 mm shaking diameter (B) side view, 530 rpm shaking frequency, d_0_ = 50 mm shaking diameter (C) top view, 750 rpm shaking frequency, d_0_ = 25 mm shaking diameter (D) top view, 530 rpm shaking frequency, d_0_ = 50 mm shaking diameter. The contact line between the bulk liquid and the glass wall is represented as blue line in the conical upper part of the shake flask and as red line in the quarter torus. The green circle represents the minimum diameter that is wetted by the rotating liquid and the orange circle marks the transition from the flat bottom to the quarter torus of the shake flask. The black square marks the center of the orbital shaking movement.
